# Addressing Older Adult Social Isolation in Various Settings With Unique Conflating Trajectories of Care Models

**DOI:** 10.1093/geroni/igaa057.2308

**Published:** 2020-12-16

**Authors:** Sharon Bowland

## Abstract

No matter the age, social isolation is common among older adults due to a number of factors. In looking at social isolation through the framework of the International Classification of Functioning, Disability and Health (ICF) model, the biopsychosocial factors intersect. Care models will be explored that involve the ICF model multi-disciplinary concepts of body functions, activities, participation and environmental factors. Developing more programs to counter isolation is critical for the health of older persons. (1) The experience of vulnerability may be overcome by banding together through assisting others or building a peer support network. (2) A holistic perspective is needed in promoting interventions that support functionality. Regular programming with body awareness and cognitive reflection is enjoyed by institutionalized older adults. (3) The role of social action and social justice in reducing social isolation is part of training social work students about the importance of culture and advocacy. (4) Collected ethnographic data found that the practice of remembrance reduces social isolation regardless of the program. Gardening and storytelling were found to be opportunities to reduce social isolation. (5) With unexpected longevity in individuals with hemophilia due to scientific advances, researchers also found shame, fear, and coping through social isolation to avoid social assumptions of health status. Care models are being explored to support this cohort. To conclude, Dr. Sharon Bowland will summarize our abstracts and discuss how they revolve around the ICF model of care that can be applied to the important social determinant of health area of social isolation.

